# The assessment of acute pulmonary embolism severity using CT angiography features

**DOI:** 10.1186/s12245-020-00272-2

**Published:** 2020-04-03

**Authors:** Azin Shayganfar, Somayeh Hajiahmadi, Mohsen Astaraki, Shadi Ebrahimian

**Affiliations:** 1 Department of Radiology, Isfahan University of Medical Sciences, Isfahan, Iran; 2 Al-Zahra Hospital, Hezar Jerib Avenue, Isfahan, Iran

**Keywords:** Pulmonary embolism, Right heart, Computed tomography, Angiography

## Abstract

**Background:**

This study was conducted to detect the association between radiologic features of CT pulmonary angiography (CTPA) and pulmonary embolism severity index (PESI).

**Methods:**

A total of 150 patients with a definite diagnosis of PE entered the study. The CTPA feature including obstruction index, pulmonary trunk size, presence of backwash contrast, septal morphology, right ventricular (RV) and left ventricular (LV) dimensions, and RV/LV ratio were examined. The severity of the PE was estimated using PESI. The association between CTPA indices and PESI was measured. Statistical analysis was conducted using the SPSS software. *P* value < 0.05 was considered as statistically significant.

**Results:**

A positive correlation was detected between the obstruction index and PESI (*r* = 0.45, *P* < 0.05). Moreover, PESI was significantly higher in patients with a more dilated pulmonary trunk (*r* = 0.20, *P* < 0.05). The backwash contrast and abnormal septal morphology were significantly more common among patients with higher PESI (*P* < 0.05). However, no significant correlation was detected between RV, LV, RV/LV, and PESI. The most predictor of high-risk PE was dilated pulmonary trunk with an odds ratio of 4.4.

**Conclusion:**

Higher Obstruction index, dilated pulmonary trunk, presence of backwash contrast, and an abnormal septal morphology can be associated with a higher PESI.

## Background

Pulmonary embolism (PE) is a type of thromboembolism that refers to obstruction of the main pulmonary artery or its branches. PE may cause various clinical presentations and even death. To reduce the morbidity and mortality caused by PE, an efficient diagnostic approach is needed for quick detection of the pathology in patients with suspected PE [1].

Computerized tomography pulmonary angiography (CTPA) is the gold standard technique in detecting PE with high sensitivity and specificity [2]. Since CTPA is the first imaging modality in detecting PE in susceptible patients, detecting predictive CTPA indices in identifying high-risk patients would be of high value to reduce the mortality rate [3].

PE can cause an increase in right ventricular (RV) afterload and dilation of RV. It may lead to a decreased LV function and output and a decrease in coronary artery supply. The reduction of blood flow to the coronary artery can cause RV dysfunction [4]. Radiologic features indicating RV dysfunction and PE severity includes RV dilation and increase the ratio of RV to left ventricular (LV) dimension, the size of pulmonary trunk, the presence of backwash contrast into inferior vena cava (IVC), and abnormal septal bowing [5–7]. The clot burden is another CTPA parameter that is shown to be associated with PE outcomes [5]. However, there are some studies reporting the clot burden score as a non-predictive index [7, 8].

Despite some studies conducted on the predictive value of CTPA indices on PE outcomes, the association between several CTPA measures of RV function and PE severity is still controversial. In this study, we aimed to detect the correlation between these indices and pulmonary embolism severity index (PESI) in patients diagnosed with PE.

## Materials and methods

This was a retrospective cohort study that was conducted on patients with a diagnosis of PE in CTPA who were referred to the Radiology Department of Al-Zahra hospital, Isfahan University of Medical Sciences, Isfahan, Iran, between April 2018 and May 2019. Patients with age > 18 years, no history of thromboembolism, thrombolytic, and anticoagulant use were included in the study.

The study was approved by the ethics committee of the Radiology Department, Isfahan University of Medical Sciences, Isfahan, Iran. The purpose of the study was described to patients, and all participants provided written consent to participation.

All images were obtained using a 128-section multi-detector CT scanner based on the standard CTPA protocol for PE (detector width 40 mm, section thickness 0.625, rotation time 0.4 sec, 120 kVp, and 380 mAs) and 20 s after administration of 100 ml contrast media at a rate of 5 ml/sec.

CTPA indexes including obstruction index, RV and LV dimension, RV/LV ratio, pulmonary trunk size, presence of backwash contrast, septal bowing, and location of embolism were evaluated. The obstruction index was measured using the Qandali score [9]. RV and LV dimensions were measured just below the atrioventricular leaflets and described as the distance between the endocardium of the septum and each ventricular wall. The pulmonary trunk size was measured in millimeter and just before the division of the artery into two main branches. A transverse CT scan was used for evaluating the septal bowing. Interventricular septal morphology was considered as normal (convex toward right ventricle) or abnormal (straight or convex toward left ventricle). Based on the site of the obstruction, embolisms were categorized into three groups including the main pulmonary artery, lobar, and segmental arteries obstruction.

Pulmonary embolism severity index (PESI) was used to estimate the 30-day outcome and was obtained by summing the patient’s age in years and point assigned for each of the 10 predictors as follows: 10 points for male sex, 30 points for a history of cancer, 10 points for a history of heart failure, 10 points for a history of chronic lung disease, 20 points for tachycardia (pulse rate ≥ 110/min), 30 points for systolic blood pressure < 100 mmHg, 20 points for tachypnea (respiratory rate ≥ 30/min), 20 points for temperature < 36 °C, 60 points for an altered mental status, and 20 points for arterial oxygen saturation < 90% [10]. According to the PESI score, patients were categorized into five classes including class I (< 65 points: very low risk), class II (66–85 points: low risk), class III (86–105 points: intermediate risk), class IV (106–125 points: high risk), and class V (> 125 points: very high risk). Then, to facilitate the statistical analyses, they were categorized into two groups including low risk (class I, class II, and class III) and high risk (class IV and class V).

All statistical analyses were performed using the SPSS software, version 21 (SPSS Inc., Chicago, IL, USA). The mean ± standard deviation (SD) value was used for the analyses of quantitative variables. Frequency and percentage were used to represent qualitative variables. Pearson correlation, independent sample *t* test, and chi-square test were used to perform the analyses. Odds Ratio (OR) was reported for the effective variables. *P* < 0.05 was considered as the statistical significance level.

## Results

In this study, 150 patients including 97 (64.7%) males and 53 (35.3%) females entered the study. The patients aged from 12 to 91 years old with a mean ± SD of 56.9 ± 17.9 years. The calculated PESI was ranged from 11 to 170 with a mean ± SD of 90.0 ± 30.1. None of the patients had hemodynamic instability and massive PE. Based on American Heart Association (AHA) classification, 90 (60%) and 60 (40%) patients had sub-massive and low-risk PE, respectively.

The mean of the obstruction index was 11.83 ± 8.40. A positive correlation was detected between PESI and obstruction index (*r* = 0.45, *P* < 0.001), and patients with high-risk PE had significantly higher PESI than those with low-risk PE (*P* < 0.05) (Table 1).

**Table 1:** The mean ± SD and frequency (%) of radiologic features in patients with low-risk and high-risk pulmonary embolism

CTA variables	Patients with Low-risk PE (*n* = 104)	Patients with high-risk PE (*n* = 46)	*P* value
Gender			
Male, *n* (%) Female, *n* (%)	65 (62.5%)	32 (69.6%)	0.404
	39 (37.5%)	14 (30.4%)	
Age	51.4 ± 16.6	69.3 ± 14.0	< 0.001
PESI	76.5 ± 21.8	120.6 ± 23.4	< 0.001
Obstruction index	9.4 ± 7.6	17.3 ± 7.6	< 0.001
RV (mm)	38.7 ± 40.7	36.9 ± 8.0	0.778
LV (mm)	39.2 ± 6.1	38.6 ± 7.6	0.595
Pulmonary trunk size (mm)	26.5 ± 3.9	28.5 ± 4.4	
Normal, *n* (%)	86 (82.7%)	24 (52.2%)	< 0.05
Abnormal, *n* (%)	18 (17.3%)	22 (47.8%)	
RV/LV ratio	1.0 ± 1.3	1.0 ± 0.3	0.857
Backwash contrast, *n* (%)			
Present	39 (37.5%)	28 (60.9%)	< 0.01
Absent	65 (62.5%)	18 (39.1%)	
Septal morphology, *n* (%)			
Normal	71 (68.3%)	20 (43.5%)	< 0.01
Abnormal	33 (31.7%)	26 (56.5%)	
Location of embolism			
Segmental artery	48 (46.2%)	4 (8.7%)	< 0.001
Lobar artery	25 (24%)	9 (19.6%)	
Main artery	31 (29.8%)	33 (71.7%)	

*PE* Pulmonary embolism, *PESI* pulmonary embolism severity index, *RV* right ventricle, *LV* left ventricle

The pulmonary trunk had a minimum size of 17.00 and the maximum size of 41.00 with a mean of 27.08 ± 4.18. Pulmonary trunk size equal to less than 29 was detected in 110 patients. The other 40 individuals had a dilated pulmonary trunk (> 29 mm). An increase in the PESI was detected with the increase in pulmonary trunk size (*r* = 0.20, *P* < 0.05). The high-risk PE was significantly more common among patients with a dilated pulmonary artery (OR = 4.4, *P* < 0.05) (Table 2).

**Table 2:** The Odds ratio of different CTA indices in predicting pulmonary embolism severity

	Odds ratio (95% CI)
Presence of backwash contrast	2.6 (1.3–5.3)
Presence of abnormal septum morphology	2.8 (1.4–5.7)
Presence of dilated pulmonary trunk	4.4 (2.0–9.4)

*CI* confidence interval

RV/LV ranged from 0.53 to 13.28 with a mean of 1.01 ± 1.07. The mean troponin and D-dimer serum levels were 78.74 ± 145.26 and 3806.42 ± 12446.37, respectively. No correlation was detected between PESI and RV (*P* = 0.778), LV (*P* = 0.595), RV/LV (*P* = 0.857), troponin level (*P* = 0.141), and D-dimer level (*P* = 0.846).

Backwash contrast into IVC was detected among 67 (44.7%) of individuals. PESI was significantly higher in patients with backwash contrast than those without a backwash contrast (*P* < 0.05) (Table 1). The presence of backwash contrast was significantly different among patients with low-risk and high-risk PE (*P* < 0.05) with a higher rate of backwash contrast in patients with high-risk PE.

Interventricular septum bowed to the right side in 91 (60.7%) of patients, while bowing to left, and straight septum was detected in 5 (3.3%) and 54 (36.0%) of individuals, respectively. The frequency of patients with abnormal interventricular septal morphology was significantly higher among patients with high-risk PE (*P* < 0.05).

PESI was significantly lower in patients with segmental artery PE than those with lobar or main artery PE (*P* < 0.05). However, no significant difference was detected between PESI in PEs located in the main and lobar pulmonary arteries (*P* = 0.233).

## Discussion

This study was conducted on 150 patients with an approved PE diagnosis to detect the CTPA indices indicating the PE with higher PESI. We found the obstruction index, the pulmonary trunk size, the presence of backwash contrast into IVC, and the abnormal septal morphology as predictive factors of higher PESI. We also investigated the dilated pulmonary trunk as the most effective predictor.

Among the signs included in the study, backwash contrast was significantly correlated with PESI. The result, in concordance with other studies, reported the presence of mentioned sign as a risk factor for mortality [11–13]. The backwash contrast into IVC is due to a right ventricular dysfunction that leads to tricuspid valve dysfunction, a decrease in forward flow from the right ventricle, and an increase of blood stasis in central veins. In addition to studies reflecting the presence of backwash contrast as a sign of RV dysfunction, some published articles have suggested this sign as an unreliable predictor [14, 15].

In this study, an abnormal septal morphology was significantly more common among patients with a high-risk PE. Interventricular septal morphology is caused by an increase of right ventricular pressure secondary to PE. It sometimes causes a leftward shift of interventricular septum that may lead to a decrease in left ventricular filling and a reduction of cardiac output, causing right ventricular wall ischemia, impaired heart function, and a poor prognosis for patients with PE [16].

In the present study, pulmonary trunk size was positively correlated with PESI. Patients with high-risk PE had significantly higher pulmonary trunk diameter. In this study, the value of 29 mm was established for the maximal value for normal pulmonary artery diameter. Also, we found that the dilated pulmonary artery had the highest OR = 4.4 in comparison with other predictors including abnormal septal morphology or the presence of backwash contrast. It suggests the idea that pulmonary trunk size can be used as a factor in prediction of patients with high-risk PE. The association between pulmonary artery diameter and short-term clinical outcomes was observed in a report of Lyhne et al. However, they did not report the pulmonary artery diameter as a strong predictor of PE outcomes [14].

There are many studies on the association between RV/LV ratio and PE outcomes. RV/LV ratio greater than 0.9 in the short axis was proposed as a sign of poor outcome and a higher 30-day risk of mortality [17–19]. However, there are some other studies reporting higher cutoff points including 1, 1.2, and 1.5 for the RV/LV ratio to be reported as an independent predictor of PE mortality [20–22]. Meinel et al. reported RV/LV diameter ratio as the strongest predictor of the clinical outcome of PTE [23]. However, we did not find any association between RV/LV ratio and PESI, which is in accordance with the report of Lyhne et al. [14]. The different results in different studies indicate that further studies are needed on this subject.

In addition to clinical symptoms and imaging features, there are many laboratory tools that are frequently utilized in patients with suspected PE, including arterial blood gas, brain natriuretic peptide, troponin, and D-dimer. These tests are not diagnostic, but they may have some prognostic values [24, 25]. In this study, we did not find any correlation between troponin, D-dimer level, and PESI. These results are in concordance with the report of Lerche et al., who stated that troponin level is not correlated to thrombotic obstruction and severity of PE [26]. However, they found a correlation between D dimer and clot burden. Meanwhile, in the present study, D dimer was not correlated with CBS and the PESI.

Although this study identified CTA indices related to higher PESI in patients with stable hemodynamic, it cannot be generalized to all patients with PE. None of the participants in this study had a massive PE. Another study is needed to identify the predictive features of CTA in patients with hemodynamic instability.

## Conclusions

In conclusion, this study suggests the CTPA indices including obstruction index, presence of backwash contrast, dilated pulmonary trunk, and presence of an abnormal septal bowing as indicators of high PESI. The physicians can use these imaging features to distinguish high-risk patients and make effective therapeutic managements.

## Data Availability

The datasets used and/or analyzed during the current study are available from the corresponding author on reasonable request.

## Abbreviations

PE: Pulmonary embolism
CTPA: Computed tomography pulmonary angiography
PESI: Pulmonary embolism severity index
RV: Right ventricle
LV: Left ventricle
